# Effects of canopy midstory management and fuel moisture on wildfire behavior

**DOI:** 10.1038/s41598-020-74338-9

**Published:** 2020-10-14

**Authors:** Tirtha Banerjee, Warren Heilman, Scott Goodrick, J. Kevin Hiers, Rod Linn

**Affiliations:** 1 Department of Civil and Environmental Engineering, University of California, Irvine, CA 92697 USA; 2 Northern Research Station, U.S. Forest Service, Lansing, MI 48910 USA; 3 Southern Research Station, U.S. Forest Service, Athens, GA 30602 USA; 4 Tall Timbers Research Station, Tallahassee, FL 32312 USA; 5 Earth and Environmental Sciences Division, Los Alamos National Laboratory, Los Alamos, NM 87545 USA

**Keywords:** Fire ecology, Forest ecology, Forestry, Atmospheric science, Atmospheric dynamics, Environmental sciences, Fluid dynamics

## Abstract

Increasing trends in wildfire severity can partly be attributed to fire exclusion in the past century which led to higher fuel accumulation. Mechanical thinning and prescribed burns are effective techniques to manage fuel loads and to establish a higher degree of control over future fire risk, while restoring fire prone landscapes to their natural states of succession. However, given the complexity of interactions between fine scale fuel heterogeneity and wind, it is difficult to assess the success of thinning operations and prescribed burns. The present work addresses this issue systematically by simulating a simple fire line and propagating through a vegetative environment where the midstory has been cleared in different degrees, leading to a canopy with almost no midstory, another with a sparse midstory and another with a dense midstory. The simulations are conducted for these three canopies under two different conditions, where the fuel moisture is high and where it is low. These six sets of simulations show widely different fire behavior, in terms of fire intensity, spread rate and consumption. To understand the physical mechanisms that lead to these differences, detailed analyses are conducted to look at wind patterns, mean flow and turbulent fluxes of momentum and energy. The analyses also lead to improved understanding of processes leading to high intensity crowning behavior in presence of a dense midstory. Moreover, this work highlights the importance of considering fine scale fuel heterogeneity, seasonality, wind effects and the associated fire-canopy-atmosphere interactions while considering prescribed burns and forest management operations.

## Introduction

Increasing wildfire impacts has been documented over the past decades^1,2^ and is projected to increase under climate change in the United States (US) and other parts of the world^1–8^. Apart from the changing patterns of precipitation, vapor pressure deficit and longer drought periods^7,9^, an increasing trend of human habitation at the wildland-urban interface (WUI) has rendered the problem of wildfire management particularly complex. The WUI is the fastest growing land-use type in the US^10^ and also poses significant wildfire threat in other countries such as Portugal. In the western US, about 50% of residential households are situated at the WUI, and flammable vegetation can come into close contact with infrastructure. This situation enhances the impact of wildfires, particularly with fast moving wildfires that escape efforts at containment during initial attack^11^.

Successful suppression of small fires, called initial attack, is critical to limiting fire size, but successful suppression is increasingly difficult in the growing WUI. Mechanical thinning and prescribed burns are offered as effective techniques to decrease fuel loads and limit fire spread into communities^12,13^. However, given the complexity of interactions between fine scale fuel heterogeneity and wind, it has been difficult to assess the success of such management operations. Thinning forests may decrease fire intensity but also increase rates of spread during critical phases of initial attack under certain conditions^14–18^. Under extreme wind events such as those experienced in Paradise, California during the Camp Fire in 2018^19^ or the south eastern Australian fires in 2019^20^, fire behaviour in fuel treatments can be affected by fire-atmosphere interactions, affecting fire suppression outcomes.

The main factors that control the rapid expansion of small wildfires are the response times of fire crew, size of fire upon initiation of suppression action^21^, and weather condition for that day. The removal of midstory and understory vegetation is targeted by managers to reduce wildfire intensity and decrease the probability of surface fire transitions to crown fire^22–25^. Thus, fuel treatment strategies focus on reduction of surface fuels, increasing the height to the live crown, decreasing crown density and a species-selective approach^12^. However, in order to assess the impact of fuel reduction treatments on fire behavior outcomes, one must consider the types of fuels, such as dead and live fuels, litter, ladder (midstory) fuels and canopy fuels associated with larger tress and their effects on fire behavior under different conditions of moisture levels^26^.

The reduction of midstory and understory vegetation does not drive fire behavior in isolation. Depending upon the seasonality and fuel conditions, midstory vegetation can increase wind drag lowering wind speeds or increase fuel moisture, which each can slow fire spread or reduce intensity. Thus, to evaluate the efficacy of fuel treatments, fuel structure alone is insufficient to understand how treatments will alter future wildfire spread and suppression success^18,27–39^. To this effect, Bessie and Johnson^40^ determined that local weather conditions, especially factors governing fuel moisture and wind speed are stronger indicators to determine fire behavior in vegetative fuel beds compared to stand age or species composition^41,42^. Keyes and Varner^43^ recognized that higher wind speeds and turbulence in the sub canopy after thinning treatment might lead to higher mid-flame wind speeds, enhanced rates of spread and erratic fire behavior. Varner and Keyes^26^ highlighted the importance of variations of fuel moisture and wind adjustment factors post fuel treatment in influencing fire spread and intensity. Moon et al.^44^ studied sub canopy wind variations under a variety of fuel structures and called for further research into fire behavior under fuel treatment scenarios which incorporates the changes in wind among other factors post treatment. A broader discussion into sub-canopy changes under fuel treatment and associated fire behavior is beyond the scope of the current paper and the interested reader is referred to Banerjee^18^ for a detailed review.

Beer^45^ identified several mechanisms through which a fire propagates within a fuel bed. He also discussed the potential influence of coherent structures such as sweeps and ejections in a vegetation canopy where a surface fire burns the understory but the canopy crown remains unburnt. The fluctuations in vertical wind velocity generated by the fire can interact with these motions and help disperse heat to the unburnt fuel elements downstream of the flame sheet. Moreover, Cheney et al.^46^ determined that while wind and dead moisture are important for grass fires, fuel load is the primary determinant of fire intensity. On the other hand, closed canopy forests with a higher moisture content and lower wind speeds can lead to lower intensity and slow moving fires^47^. Additionally, changing stand structure by thinning can lead to reduced torching and crowning potential^48,49^. Contreras et al.^50^ used light detection and ranging (LiDAR) mapped forest structure data to characterize the role of vegetation connectivity and thinning operations (that reduce connectivity) in the context of crown fire potential. White et al.^51^ and Davies et al.^52^ determined that the flammability of surface fuels are also important in governing fire severity. The type of ignition is another factor that sets up the initial condition for fire propagation. Keeley and Syphard^53^ identified the major wildfires in California from 2003 to 2018 and deduced that the fire regimes can be either identified as fuel dominated or wind dominated. In either case, the complex fire-fuel-atmosphere interaction is of critical importance.

Fuel moisture patterns in humid environments are more complex than previously assumed^54^, and environmental conditions during a wildfire event may have non-linear treatment effectiveness outcomes. Finney et al.^55^ identified the importance of studying turbulent flows associated with fuel structures and fuel moisture, especially how they contribute to buoyancy production and flow instabilities, but as yet, these complex coupled fire-atmospheric dynamics have not been applied to the question of fuel treatment effectiveness on initial attack success.

Computational fluid dynamic (CFD) modeling approaches are capable of representing the non-linear feedback between changes in forest structure, complex in stand flows, and fire behavior outcomes^56–64^. Pimont et al.^65^ used FIRETEC to study the effect of different fuel treatments in the landscape on fire behavior. Linn et al.^66^ used FIRETEC to model wind fields and fire propagation following bark beetle outbreaks. Kiefer et al.^67^ studied the detailed budget of turbulent kinetic energy during a low intensity fire and investigated the sensitivity of mean and turbulent flows to canopy density as well as atmospheric stability using the ARPS-CANOPY model. Another series of studies^68–72^ conducted detailed turbulence measurements during grass fires and surface understory fires using high frequency micrometeorological measurements from the FIREFLUX campaigns and two New Jersey Pine Barrens fire experiments.

However, there remains uncertainty regarding the complicated fire–atmosphere interaction in the canopy sub layer in presence of midstory vegetation of different densities, which governs fire spread in treated vs. untreated fuels. In this work, simulations using FIRETEC are used to address the following questions:

- What are the driving factors governing fire behavior under different levels of midstory management and fuel moisture?
- What are important factors leading to torching and crowning?
- How to characterize turbulent transport of momentum and energy to explain fundamental differences in fire behavior?

To answer these questions, specifically six sets of simulations are conducted. The cases are described in more detail in the methods section. The simulation cases are called dry no midstory (DN), dry sparse midstory (DS), dry dense midstory (DD), moist no midstory (MN), moist sparse midstory (MS) and moist dense midstory (MD), respectively. For brevity, these abbreviated forms will be used for further discussion. It is also important to note that the vegetation phenology in this study is driven by seasonality rather than atmospheric conditions.

**Figure 1:**
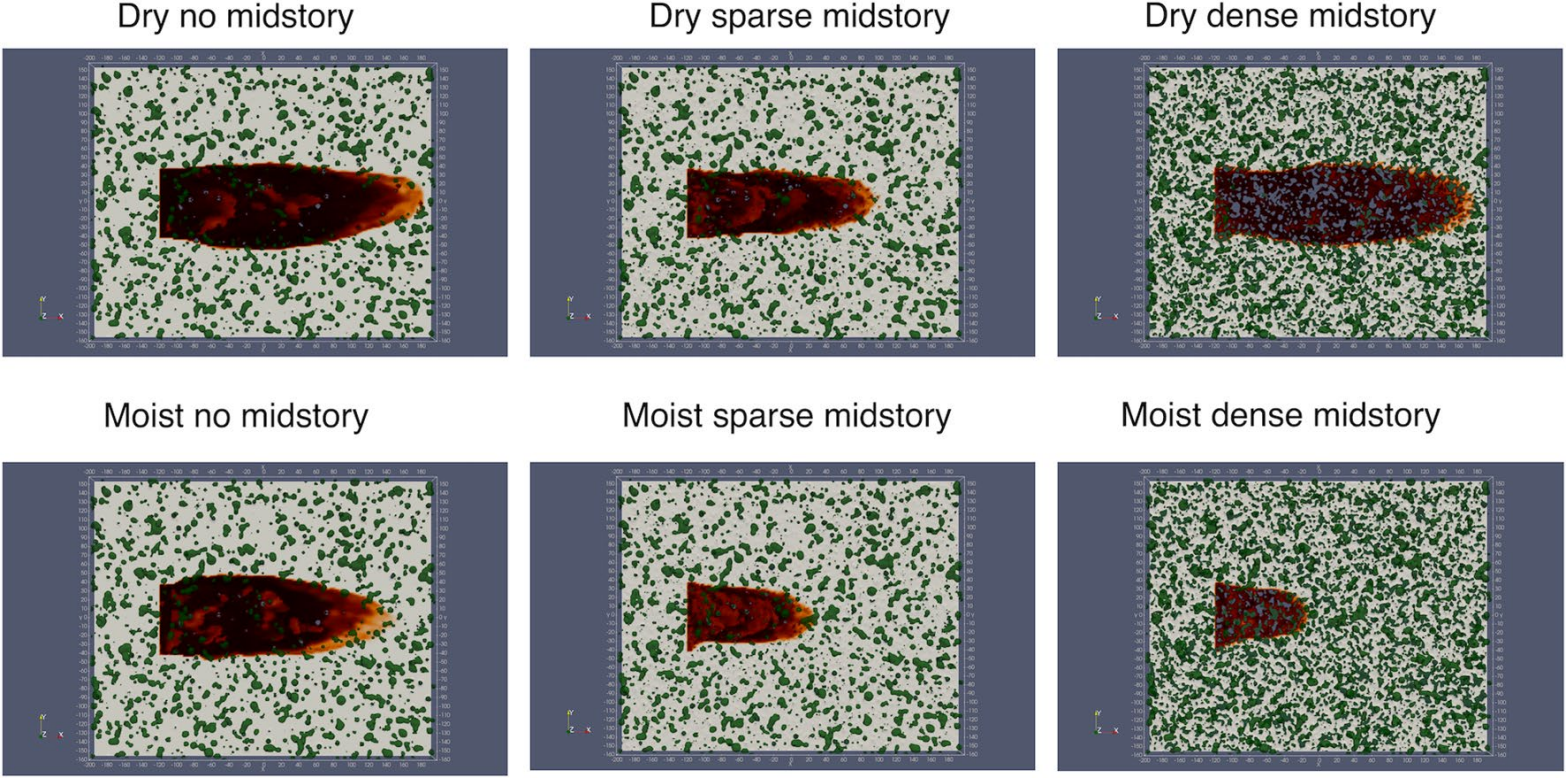
Burnt area after 520 s for different cases. Green depicts midstory vegetation, the light yellow shade depicts the ground surface covered with grass and litter and the dark color depicts burned area.

## Results

### Burnt area

**Figure 2:**
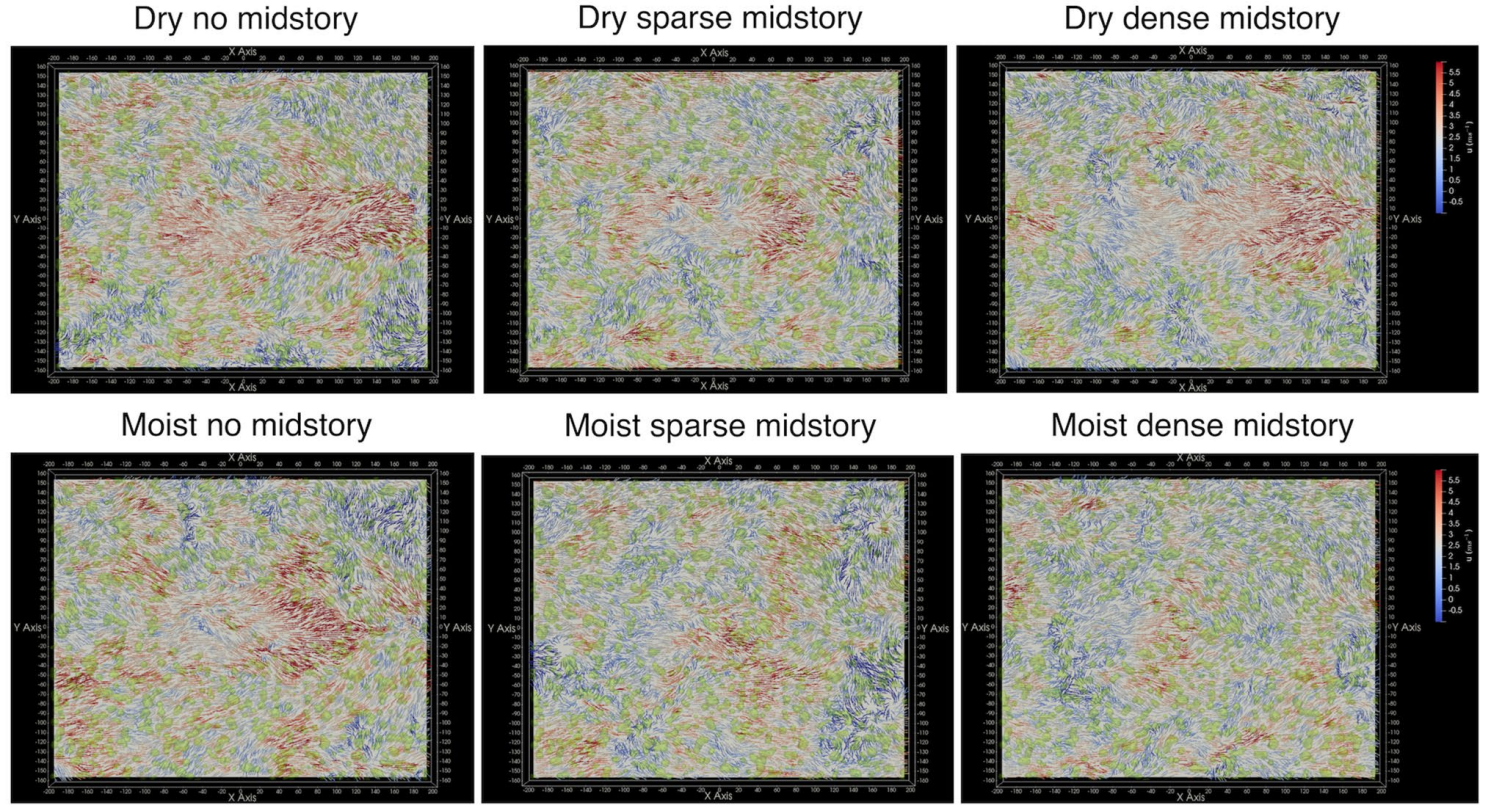
Sub canopy wind field ($x-y$ plane at 7 m height) with arrows colored by instantaneous streamwise velocity component (*u*) after 520 s for the six scenarios.

Figure 1 shows the burnt area after 520 s of fire propagation. Since the initial and boundary conditions are same for all the simulations, the differences in fire spread are entirely due to differences in canopy structure among the different cases. For the dry scenario, the DN case and the DD case have burnt areas of similar sizes, although the burn scar shapes are slightly different. The DS case has a smaller and thinner burn area. For the moist scenario, the MN case has the largest burn area. The size of the burn area is smaller for the MS and smallest for the MD case. Figure 1 highlights a crucial factor—namely the competing influences of fuel availability, fuel moisture and wind effects. Under dry conditions, the availability of dry fuels in the DD case creates a strong head fire. However, the DN case is characterized by a stronger wind field inside the canopy due to lower vegetative drag. This indicates that there is likely a threshold effect, where either the fuel availability or the wind effects dominate. The DS case falls in the intermediate regime, and consequently has a smaller burn scar. Under moist conditions, the higher fuel moisture dampens the effect of fuel availability on fire propagation, and thus the wind effect dominates. This fact is highlighted in Fig. 2, which plots the horizontal ($x-y$) wind field at 7 m height, with velocity vectors colored by the streamwise velocity component (*u*). As observed, the no midstory case has higher wind speeds compared to the other cases due to lower vegetative drag. A more quantitative understanding of the role of turbulence will be discussed in a subsequent section.

### Fire intensity, fire spread and fuel consumption

**Figure 3:**
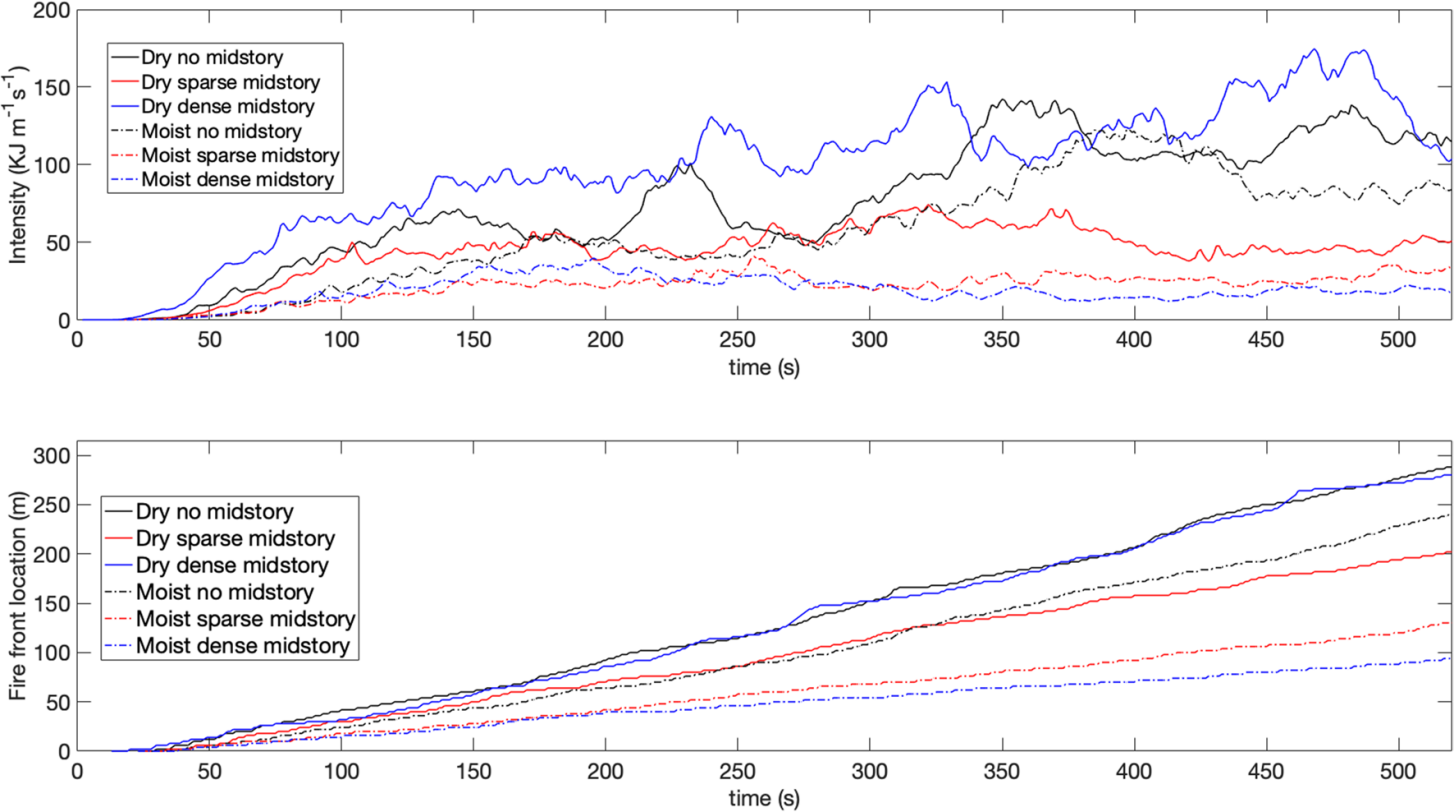
Burning intensity (top) and fire front location with respect to time (bottom) for different cases. The black lines indicate non midstory cases, the red lines indicate the sparse midstory cases and the blue lines indicate the dense midstory cases. The solid lines indicate dry scenarios and the dash dotted lines indicate moist scenarios.

Figure 3 shows burning intensity (top panel) and the location of the fire front (bottom panel) with respect to time. As observed, the DD case has the highest intensity because of elevated fuel availability among all cases. Interestingly the DN case burns more intensely compared to the DS case, highlighting the critical interaction between fuel availability and wind effects. The moist cases generally showed reduced fire intensity than the dry cases as expected. The differences among the fire intensities observed within the moist cases are smaller than those among the dry cases. This implies the absence of any critical behavior feedback for the moist cases with respect to the competition between wind effects and fuel availability and the dominance of wind effects for the moist scenarios.

**Figure 4:**
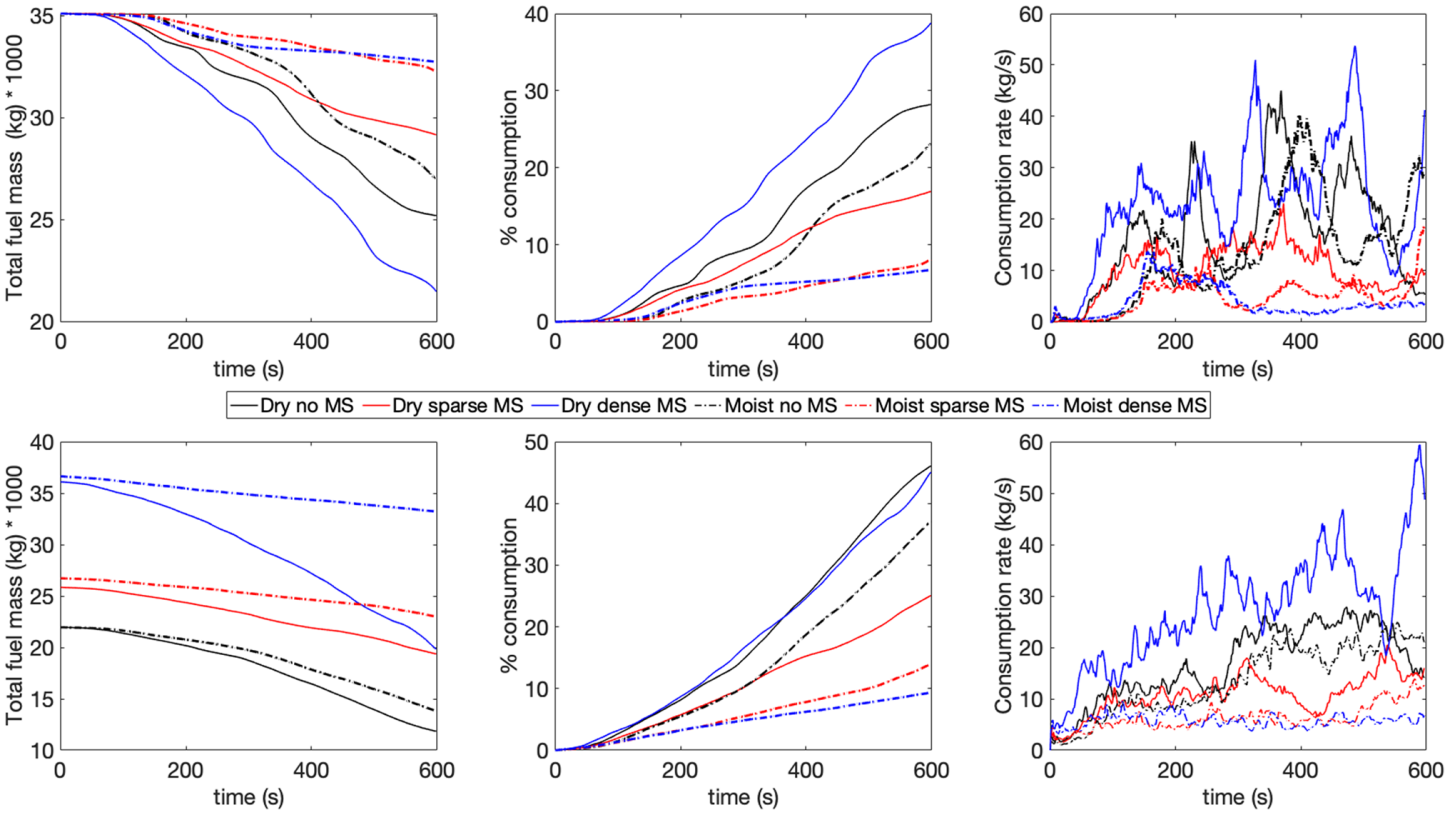
Actual fuel amount remaining over time (left), percentage consumption (middle) and rate of consumption (right) of fuel elements with respect to time for different cases. Line styles are same as Fig. 3. The top row shows the quantities for the overstory and the bottom row for the midstory and understory combined.

Interestingly the fire front propagation spread rate follows the trend of the intensities. However the difference between the cases do not follow the trend in differences of fire intensity. The DN and DD cases have similar spread rates and these two cases show the fastest fire front propagation, although the DN case is slightly faster. The reason for this behavior is likely the same—one dominated by dry fuel availability and the other by strong winds. The effect of the wind prevails for the moist cases as well and the MN case is faster than the MS and MD cases. To summarize, it can be stated that fire intensity is strongly governed by fuel availability and fire propagation speed is governed by wind effects. However, fuel moisture significantly reduces both fire intensity and rate of spread. This is partly because fire induces its own wind environment as well. However, under drier scenarios the effect of vegetative drag becomes more evident.

Figure 4 shows the consumption of canopy overstory fuels (top panels) and canopy midstory and understory fuels (bottom panels) after 520 s. The left panels shows the actual amount of fuel remaining over time in metric tons (kg multiplied by 1000) for the entire domain (12.8 ha), the middle panels show the percentage consumption and the right panels show the rate of consumption per second. Any vegetation below 5 m height is considered midstory and understory vegetation. Note that all cases start with the same amount of overstory fuel (35 metric tons). As the leftmost bottom panel shows, the dense midstory cases have about 35 metric tons of midstory and understory fuels, although the moist case has slightly more fuel, as expected, since the deciduous midstory seasonally loses leaf biomass. The sparse midstory case has about 27 metric tons midstory and understory fuels in the moist season and about 26 metric tons in the dry season. The no midstory case has about 22 metric tons of understory fuels, and that amount does not vary in the dry and moist seasons.

A few observations can be made from Fig. 4:

- **Net consumption** The net consumption for the overstory is highest for the DD case, about 40%. The DN case has a similar consumption rate—about 30%. Interestingly, both the dry dense and DN cases have about 45% consumption for the mid and understory fuels. This indicates that while crowning happens for both these cases, driven by the dry fuels and higher winds, the presence of midstory and understory fuels influence how much overstory fuel the crown fire consumes. Moreover, the MN case has about 25% consumption for the overstory fuel and 35% consumption for the mid and understory fuels. The fact that the MN case has more consumption than the DS case is also highlights that wind effects can dominate over moisture effects.
- **Difference based on fuel moisture** The dry fuels are always consumed more than moist fuels, as expected, both for overstory and understory vegetation. The difference between dry and moist scenarios is most prominent for the dense midstory cases—about 35% more overstory and about 40% more mid and understory fuels are consumed in the dry condition compared to the moist condition, highlighting the importance of fuel moisture and seasonality in fire behavior. This difference is strikingly lower for the no midstory cases—about 5% for both overstory and mid/understory fuels. This indicates that when wind effects are more prominent, seasonal changes in moisture are less important. For the sparse midstory cases, this difference is about 10% for both overstory and mid/understory fuels, highlighting the fact that fire behavior is partially dominated by both wind and moisture effects.
- **Rate of consumption** The rate of consumption with respect to time closely follows the trends of actual consumption. However it is important to note that the rate curve is not uniform and is highly variable in time. This non uniformity is more conspicuous for the overstory fuel compared to the midstory and understory fuels. This is due to the complex nature of the turbulence and combustion phenomena across a strongly heterogeneous canopy fuel complex.

To understand the role of turbulence inside the canopy in more detail, virtual sensors were placed at different locations on the domain, so turbulent statistics could be calculated. In section, time series of such turbulence statistics are presented.

**Figure 5:**
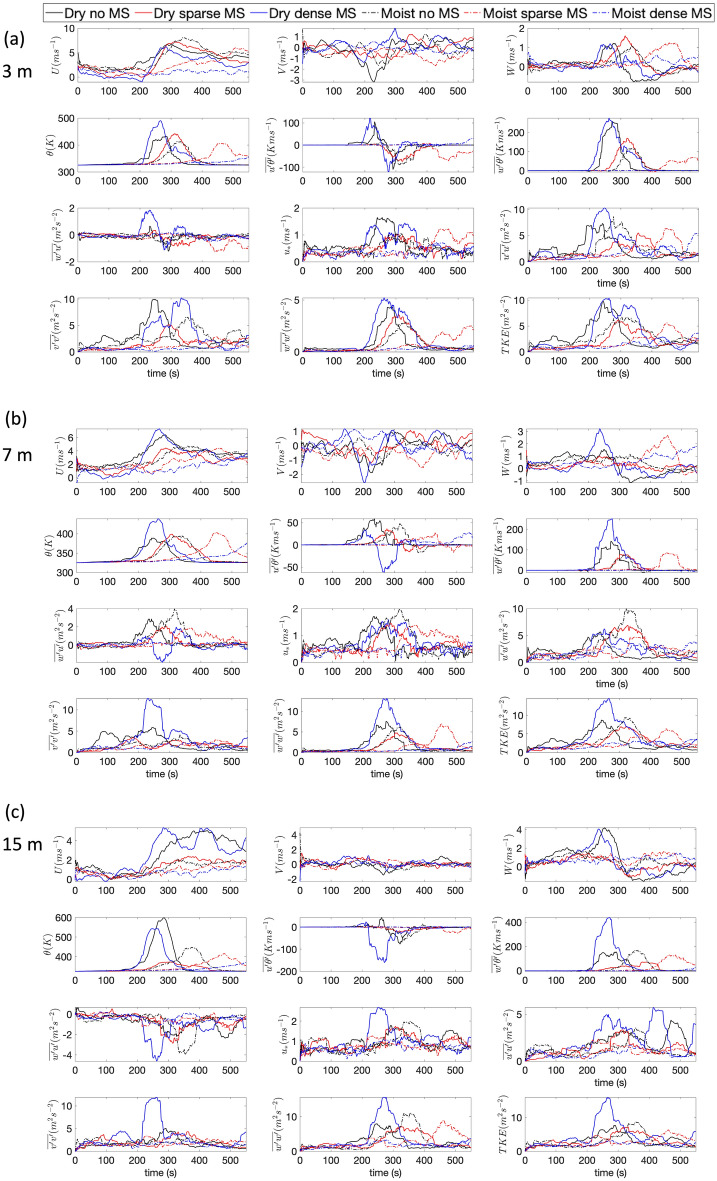
Moving average of turbulence statistics at (**a**) 3 m height, (**b**) 7 m height and (**c**) 15 m height at the domain center. Line styles are same as Fig. 3. *U* is the mean streamwise velocity, *V* is the mean cross stream velocity, *W* is the mean vertical velocity, $\theta$ is potential temperature, $\overline{u^{\prime }\theta ^{\prime }}$ and $\overline{w^{\prime }\theta ^{\prime }}$ are sensible heat flux along the streamwise (*u*) and vertical (*w*) directions (the primed quantities are fluctuations from the mean). $\overline{w^{\prime }u^{\prime }}$ is the vertical momentum flux, $\overline{u^{\prime }u^{\prime }}$, $\overline{v^{\prime }v^{\prime }}$ and $\overline{w^{\prime }w^{\prime }}$ are velocity variances in *u*, *v* and *w* directions respectively. TKE is turbulent kinetic energy and defined by one half of the summation of $\overline{u^{\prime }u^{\prime }}$, $\overline{v^{\prime }v^{\prime }}$ and $\overline{w^{\prime }w^{\prime }}$.

### Characterization of wind and turbulence

**Figure 6:**
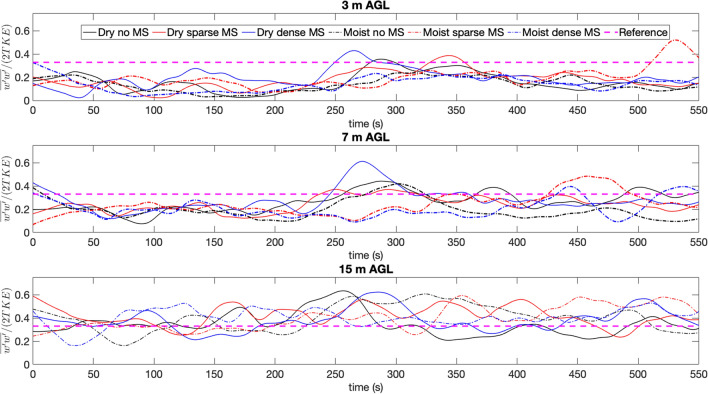
Time variation of 1 min averaged values of $\overline{w^{\prime }w^{\prime }}/(2*TKE)$ showing turbulent anisotropy. Line styles are same as Fig. 3. Dashed magenta line shows the value of 0.33 for isotropic turbulence.

Figure 5a shows 1 min moving average means of several quantities at 3 m height at the domain center. Figure 5b shows the same at 7 m and Fig. 5c shows the same for 15 m height. These three locations are chosen so the variations in turbulent quantities and fluxes can be shown with time as the fire approaches the domain and with height so the dynamics inside the canopy can be probed in a much more detailed and quantitative way. Several observations can be made from Fig. 5a–c:

- **Mean streamwise velocity** (*U*) Because of higher vegetative drag, the *U* velocities are higher for the cases with no midstory and sparse midstory compared to the dense midstory case before the fire reaches the center of the domain. The DD case also records negative *U* velocities at 3m height before the fire starts influencing the velocity field. The velocities for all cases are centered around 1–3 $\mathrm {ms}^{-1}$ before the influence of the fire, with the dense midstory cases (dry and moist) recording the lowest *U* velocities. Once the fire starts influencing the velocity field, the *U* velocities for the six different cases diverge widely. Still, the no midstory cases report the highest *U* velocities—about 6–8 $\mathrm {ms}^{-1}$. However, when the fire reaches the domain center, the DN case records a strong *U* velocity even at 7 m height (7 $\mathrm {ms}^{-1}$ ). This is likely due to also the loss of vegetation due to burning, which reduces the drag force when the fire passes a particular region. The DN case also records strong *U* velocities at 7 m height, both before and during the fire front passage. At 15 m, the high canopy drag results in lower velocities for all the cases except for the DD case and the DN case. The high velocity for the DD case is partly due to loss of fuel by burning and partly due to high turbulence due to buoyancy. Note that *U* is generally higher for all cases the the canopy mid-story and understory compared to the canopy crown region where the drag is much higher.
- **Mean cross stream velocity** (*V*) Although the inflow boundary conditions prescribe a zero *V* component, it becomes finite as the flow field is influenced by the fire. Before the fire influences the domain, the *V* velocities are centered around zero. However they start to diverge as the fire gains strength. As noticed in Fig. 2, there is a significant cross flow component as the cold wind wraps around the fire and entrains the burnt area behind the top flank of the fire. This *V* component also experiences drag and thus the no midstory case has the highest ($-\,3\,\mathrm {ms}^{-1}$) *V* velocity at 3 m height. Interestingly, the DD case also picks up a strong *V* component, probably influenced by cold air entrainment due to high intensity burning. At 7 m height, the finite *V* component still persists. However, at 15 m, the *V* velocity component is almost zero, due to the strong drag effects of the vegetation crown.
- **Mean vertical velocity** (*W*) At 3 m height, the cases with no and sparse midstory has strong vertical updrafts (1.5–2 $\mathrm {ms}^{-1}$). This can be attributed to lower drag forces, as dry and moist scenarios for these two cases record similar *W* components as well. Interestingly, the DD has strong updrafts but the MN case does not. This indicates that the buoyancy generated due to intense burning of dry fuels is responsible for the updrafts. Note that the strong updrafts are followed by downdrafts (order of 0.5 $\mathrm {ms}^{-1}$), as the cold air entrains the burnt area behind the fire front. At 7 m, the DD case reports even higher updrafts (3 $\mathrm {ms}^{-1}$) and a stronger downdraft ($-\,1\,\mathrm {ms}^{-1}$) after the fire front moves past the virtual sensor. This is likely due to the presence of dense dry midstory which burns vigorously, and supported by the fact that under moist conditions, the dense midstory case has lowest *W* components. This effect is dominated by stronger drag forces. Interestingly, the sparse moist midstory case also has strong updrafts ($2.5\,\mathrm {ms}^{-1}$), likely due the nonlinear combination of drag and buoyancy effects. At the crown level (15 m height), both the dry dense and DN cases record strong updrafts (4 $\mathrm {ms}^{-1}$), due to effects described previously.
- **Potential temperature** ($\theta$) at 3 m height, the air temperature reaches the highest level (500 K) for the DD case. The no midstory and sparse midstory cases reach slightly lower temperature (about 375 K). The dry cases reach higher temperatures than the moist cases, as expected. The only exception is the MS case (about 300 K). This also explains the previous observations regarding the high updrafts for the dry midstory cases. At 7 m height, the difference among the cases become more prominent. The DD case reaches a peak temperature of about 450 K and the other cases reaches around 400 K when the fire reaches the sensor. At the crown level of 15 m, the dry dense and DN cases reach high temperatures of about 550–600 K. The other cases still record about 400 K.
- **Streamwise sensible heat flux** ($\overline{u^{\prime }\theta ^{\prime }}$) This term can also be called the kinematic advective heat flux along the streamwise (*x*) direction and is a measure of horizontal heat transfer along the mean flow direction at any level. A positive heat flux should indicate advective heating of the fuel elements in the direction of the fire spread. This is why the peaks for $\overline{u^{\prime }\theta ^{\prime }}$ occur before the spike in temperature as the fire approaches the sensor. The DD case records the highest $\overline{u^{\prime }\theta ^{\prime }}$ at 3 m height (about 110 $\text {K ms}^{-1}$), followed by the DN case. After the fire passes, the $\overline{u^{\prime }\theta ^{\prime }}$ becomes negative, indicating that the fuel elements are cooling down. At 7 m height, the DN case records more advective heating compared to the DD case. However, the magnitude of $\overline{u^{\prime }\theta ^{\prime }}$ is lower at 7 m, about 50 $\text {K ms}^{-1}$ for the DN case. This can be attributed to higher amount of fuel availability at this level. Interestingly, the moist cases record $\overline{u^{\prime }\theta ^{\prime }}$ of a similar order of magnitude. This indicates that advective heating at the midstory level in the model is dominated by wind and drag, not moisture. At the crown height of 15 m, the heating effects are even smaller than the midstory and understory levels. However, there is strong cooling effect, probably attributed to the crowning behavior at this level.
- **Vertical sensible heat flux** ($\overline{w^{\prime }\theta ^{\prime }}$) A positive value of $\overline{w^{\prime }\theta ^{\prime }}$ represents an upward flux of warm air due to buoyancy and should be interpreted as a measure of the strength of the buoyant flame dynamics. The presence of a fire generates buoyancy driven turbulence, which should result in a strongly positive $\overline{w^{\prime }\theta ^{\prime }}$. The dry cases register higher values of vertical sensible heat flux compared to the moist cases. At 3 m, both the DD and DN cases record similar $\overline{w^{\prime }\theta ^{\prime }}$, about 250 $\text {K ms}^{-1}$. The DS case records about 175 $\text {K ms}^{-1}$. At 7 m, the trends remain similar, although the DD case $\overline{w^{\prime }\theta ^{\prime }}$ values (about 250 $\text {K ms}^{-1}$) are nearly twice as large as the the DN case. This behavior is an indicator of laddering as the fire climbs up towards the crown. The MN case has lower $\overline{w^{\prime }\theta ^{\prime }}$ at 7 m height (about 70 $\text {K ms}^{-1}$) which indicates that the sensible heat flux is definitely impacted strongly by fuel moisture. However, the dry sparse and moist sparse cases record similar $\overline{w^{\prime }\theta ^{\prime }}$ at 7 m, which means that under sparse conditions, moisture effects are less prominent. At the crown height of 15 m, the DD case records a much stronger $\overline{w^{\prime }\theta ^{\prime }}$ of about 400 $\text {K ms}^{-1}$. while the other cases remain similar to the 7 m level. This increasing $\overline{w^{\prime }\theta ^{\prime }}$ with height for the DD case is an indicator of crown fire behavior.
- **Vertical momentum flux** ($\overline{w^{\prime }u^{\prime }}$): The parameter $\overline{w^{\prime }u^{\prime }}$ represents the vertical flux of horizontal momentum and its value should be negative inside the vegetation canopy in regular atmospheric boundary layer flow as the canopy absorbs momentum from the flow and acts as a momentum sink. The value of $\overline{w^{\prime }u^{\prime }}$ should also change as the canopy is consumed by the fire, which changes canopy drag. However, as the presence of fire create a strong buoyancy driven updraft, it can create a positive momentum flux in the canopy sublayer. At the 3 m height, all the cases record negative $\overline{w^{\prime }u^{\prime }}$. However, as the fire passes the virtual sensor, the DD case records a strong positive $\overline{w^{\prime }u^{\prime }}$. However, as the fire passes the sensor, cool air rushes in a negative $\overline{w^{\prime }u^{\prime }}$ is recorded because of strong drag effects. The DN case also shows a similar behavior although the peak values are much lower. At 7 m height, the no midstory and sparse midstory cases, (both dry and moist) records strong positive $\overline{w^{\prime }u^{\prime }}$. Higher drag at this level reduces $\overline{w^{\prime }u^{\prime }}$ for the DD case. Interestingly, at 7 m level, the burning of midstory fuels still generate upward fluxes of momentum. At the 15 m level, the upward flux of momentum is small, indicating that the crown region absorbs the locally generated upward momentum flux and the fire does not ‘leak’ additional momentum into the atmospheric surface layer above the canopy. Moreover, at the crown height of 15 m, the canopy also absorbs momentum from the overlying air mass perturbed by the fire and the DD case absorbs most of it even when the fire is burning strongly. Another interesting fact is that the momentum flux is not very sensitive to moisture effects.
- **Friction velocity** ($u_*$) The friction velocity is computed as
  1 $$ u_*={(\overline{u^{\prime }w^{\prime }}^2+\overline{v^{\prime }w^{\prime }}^2)}^{1/4}, $$
  where $v^{\prime }$ denotes cross stream velocity fluctuations and $u_*$ represents the net magnitude of wind shear stress at a particular height. In regular atmospheric turbulence, $u_*$ can range between 0.1 $\mathrm {ms}^{-1}$ to 0.5 $\mathrm {ms}^{-1}$. At the 3 m height, $u_*$ is indeed at that range for all cases, until the fire enhances the magnitude of the turbulence locally. This increase of $u_*$ is observed for all cases during fire front propagation, which is associated with strong vertical motions close to the flame associated with a ‘chimney effect’^69^, resulting in higher turbulent stress. At 3 m height, the DN case records $u_*$ around 1.6 $\mathrm {ms}^{-1}$ as the fire passes the sensor. The DD case records a slightly lower $u_*$ but of similar order of magnitude during fire passage. The sparse cases also report a $u_*$ about 1.2 $\mathrm {ms}^{-1}$. At 7 m level, $u_*$ is higher for all the cases, while the no midstory cases reach magnitudes around 2 $\mathrm {ms}^{-1}$. The amount of moisture does not have much impact in the magnitude of shear stress and potentially is more strongly driven by the amount of fuel present and at the rate fuel is removed by fire. $u_*$ returns to pre-fire magnitudes after the fire passes, in spite of ongoing smoldering. Interestingly, at crown height, the DD case has the highest magnitude of $u_*$ (around 2.6 $\mathrm {ms}^{-1}$), likely due to buoyancy generated turbulence during the fire. Before and after the fire passage, the dense midstory case has much lower magnitudes of $u_*$.
- **Turbulent kinetic energy (TKE):** The turbulent kinetic energy is computed as
  2 $$ TKE=\frac{1}{2}\left( {\overline{u^{\prime }u^{\prime }}^2+\overline{v^{\prime }v^{\prime }}^2+ \overline{w^{\prime }w^{\prime }}^2}\right) , $$
  where $u^{\prime }$, $v^{\prime }$ and $w^{\prime }$ are fluctuations from the 1 min moving averaged mean. Figure 5 shows the individual components of TKE as well as the net TKE for the three heights and for the six different cases. At 3 m height, before the fire, the DN case has higher magnitudes of TKE, about 2.5 $\mathrm {m}^2{\mathrm{s}}^{-2}$, while the DD case has slightly lower TKE close to the surface. This indicates the effect of fuel drag. As the fire passes, the TKE for all cases increases significantly, from 0.5-2.5 $\mathrm {m}^2{\mathrm{s}}^{-2}$ to about 10 $\mathrm {m}^2{\mathrm{s}}^{-2}$. The DD case records the highest TKE in this range. Interestingly, this TKE rise has two components. First, the large rise is contributed by $\overline{u^{\prime }u^{\prime }}$, during intense burning between 200 and 300 s. Next, a strong rise is recorded for $\overline{v^{\prime }v^{\prime }}$, between 300 and 400 s, which leads to the net TKE peak that lasts between 200 and 400 s. This rise is associated with strong crosswind flows that wrap around the fire. The $\overline{w^{\prime }w^{\prime }}$ component shows the contribution from buoyancy driven turbulence, which is vertical in direction. The DD case records the highest $\overline{w^{\prime }w^{\prime }}$ as well due to most intense burning, followed by the DN and DS cases. The moist cases record lower $\overline{w^{\prime }w^{\prime }}$ which is expected, as the intensity of burning is less. However, the patterns of $\overline{u^{\prime }u^{\prime }}$ and $\overline{v^{\prime }v^{\prime }}$ are more complicated as they are dependent of vegetative drag and how fuel is removed with burning, which also dictates the nature of the wind as it rushes to the upstream of the fire flank as the fire passes the area. The net TKE contains all these combined effects. After fire front passage, TKE values return to their pre-fire-front-passage values. Another interesting observation is that even during fire front passage, the relative contributions of $\overline{u^{\prime }u^{\prime }}$, $\overline{v^{\prime }v^{\prime }}$ and $\overline{w^{\prime }w^{\prime }}$ remain similar, i.e $\overline{w^{\prime }w^{\prime }} \approx 0.5 \overline{u^{\prime }u^{\prime }}$ and $TKE \approx \overline{u^{\prime }u^{\prime }} \approx \overline{v^{\prime }v^{\prime }}$ in terms of magnitude, which is also observed in regular atmospheric turbulence^73^. At 7 m height, the net TKE follows similar patterns, although the DD case records about 15 $\mathrm {m}^2{\mathrm{s}}^{-2}$ during fire passage, while the DN case is still at 10 $\mathrm {m}^2{\mathrm{s}}^{-2}$. This contribution is mainly due to buoyancy effects and cross stream velocity components, as the $\overline{w^{\prime }w^{\prime }}$ increases significantly at this height, about 15 $\mathrm {m}^2{\mathrm{s}}^{-2}$, due to higher fuel availability. At this height, the contribution to TKE from $\overline{u^{\prime }u^{\prime }}$ is rather small, because of higher fuel drag. The no midstory and sparse midstory cases have higher $\overline{u^{\prime }u^{\prime }}$ at this height. At the crown height of 15 m, the trends are similar to the midstory level. Another point to note here that the level of vertical turbulence can also set the boundary conditions for spotting potential, which can launch firebrands aloft. These embers and other burning particles can get transported by the turbulent wind aloft the canopy sub layer and create spot fires ahead of the fire front.
- **Isotropy** Another factor associated with TKE is isotropy. Figure 6 shows the time variation of $\overline{w^{\prime }w^{\prime }}/(2*TKE)$ for all 6 cases, for the three heights 3 m, 7 m and 15 m. For isotropic turbulence, this value should be 0.33^71,74^. If this value is $< 0.33$, it indicates that the horizontal component of the TKE ($\overline{u^{\prime }u^{\prime }}+\overline{v^{\prime }v^{\prime }}$) strongly dominates over the vertical component $\overline{w^{\prime }w^{\prime }}$. Before the fire front passage, all cases exhibit strong anisotropy, however, the anisotropy is stronger (further away from 0.33) close to the ground surface and is more isotropic at crown height. During fire front passage, the strong buoyancy effects enhance $\overline{w^{\prime }w^{\prime }}$ and increases isotropy for all cases and all levels, especially for the DD case.

There are few instances in the literature that have reported fine-scale turbulent quantities in such detail as discussed above, especially in the context of wildfires spreading in a vegetative environment. However, Clements et al.^69^ had conducted field experiments (FIREFLUX) which collected high frequency turbulent data on a micro-meteorological tower at four heights (2 m, 10 m, 28 m, 42 m) in the path of a grass fire. This allowed the authors to examine mean and turbulent quantities before, during and after fire front propagation. Clements et al.^69^ used 1 min moving averages to look at the evolution of flow statistics as the fire passed the tower, which is the strategy used in this work as well. They observed friction velocities ($u_*$) in the range of 2.5–3 $\mathrm {ms}^{-1}$ at 2 m height and 3–3.5 $\mathrm {ms}^{-1}$ during fire front passage. The $u_*$ observed in this study has a similar order of magnitude. Moreover, they observed TKE in the range of 6–12 $\mathrm {m}^2{\mathrm{s}}^{-2}$ which is also the range observed in this study. Heilman et al.^74^ reported peak TKE values of 5–20 $\mathrm {m}^2{\mathrm{s}}^{-2}$ during two burns at the New Jersey Pine Barrens (forest environment). Heilman et al.^71^ reported similar trends of turbulence anisotropy (0.10–0.20) during these burn operations. The buoyancy flux, defined as $B_f=g/\theta *\overline{w^{\prime }\theta ^{\prime }}$ are also on the order of 0.5–1 $\mathrm {m}^2{\mathrm{s}}^{-3}$ similar to the magnitudes reported by^69^. However, the difference between fuel characteristics (grass in the case of^69^, forest vegetation in this case) precludes further one to one comparisons.

## Discussion

In this work, we investigated the drivers of wildfire spread following linear ignition in the context of active fuel reduction treatments. We further examined how clearing midstory vegetation alters fire behavior through changes in canopy drag and the interdependence on fuel moisture. A line ignition was used to investigate the fundamental processes in fire behavior, with the application of these simulations addressing potential fire size on initial attack success. Simulations in this study were conducted using HIGRAD/FIRETEC to observe bulk fire behavior indicators such as fire intensity, fire spread rate and fuel consumption.

The generally expected trend was found with the DD case, which produced the highest fire intensity. Interestingly, under dry conditions, the gradual lowering of midstory density did not yield monotonically decreasing trends of fire intensity. Up to a level of midstory thinning, termed sparse midstory in this study, the fire intensity and rate of spread were reduced. However, under the DN case, where most of the midstory and understory vegetation was thinned, the wind speed and turbulence levels were enhanced such that the rate of spread increased and was often higher than the DD case. More interestingly, the fire intensity actually increased compared to the DS case with the absence of midstory but lower than the DD case. The enhanced wind speed, turbulence and the resulting augmented sensible heat flux were partly responsible for this behavior, which is seemingly counter intuitive as the DS case was characterized by more fuels.

This behavior further highlight the trade off between fuel availability and wind effects. Under the DN case, the wind effects dominated as the canopy drag was low. Under the DD case, the fuel effect dominated as there is simply too much dry fuel. In the sparse case where both the fuel effects and wind effects were moderated, both fire spread and fire intensity were reduced. This trade off offers another perspective on the practice of fuel treatments with prescribed fires. Under dry conditions, both no thinning and excessive thinning can lead to high amount of fuel consumption. Whereas a moderate degree of thinning can lead to lowered consumption for both overstory and understory vegetation. Hence burn managers might consider this trade-off on consumption as well as the consequences on fire intensity and rate of spread when conducting cost-benefit analyses of a prescribed fire.

Under seasonably moist conditions, these trade-offs were absent and wind effects dominated the fire, with the fire being slower and less intense with higher fuel moisture. Higher winds drove the fire faster under moist conditions. To understand the physical mechanisms behind this behavior, detailed analyses were conducted by collecting data on virtual towers at different locations. Time series of flow quantities such as mean velocities, potential temperature and turbulent statistics such as sensible advective and buoyant turbulent heat fluxes, turbulent stress (momentum flux), friction velocity (a measure of shear stress) and turbulent kinetic energy and its components were plotted at the domain center, at three different heights, close to the surface, at midstory height and at crown height.

These emergent picture of the consequences of nonlinear fire atmosphere interaction in the fine scales on fire behavior is also consistent with new insights coming out the analyses of a large number of recent fires in California^53^, which were identified to be either fuel dominated or wind dominated. Consequences of long term fire suppression policy, silvicultural practices, grazing and timber harvesting practices can alter the fire regime in either directions. For the fuel driven fires, altering fuels can offer bottom up controls as hypothesized by Keeley and Syphard^53^ and clearly demonstrated in this work. It is also important to recognize that when extreme synoptic scale wind events (such as Santa Ana winds) dominate fire behavior, fuel treatments are hardly a limiting factor and those cases are beyond the scope of the current manuscript. However, mastication and thinning operations can establish a higher degree of control even on fires on shrub type ecosystems under lower wind events^75,76^. Future research will attempt to establish the limits of these top down and bottom up controls on wildland and prescribed fires, as well as their interaction with complex terrain.

Nevertheless, a physical understanding of canopy-fire atmosphere in such detail as explored in this work can help burn managers design treatments to alter fire behavior. Moreover, the patterns of fire spread within treatment zones during initial attack will depend heavily on interacting factors of canopy-induced winds, fuels moisture, and loading. The suite of conditions under which desired fire behavior can increase suppression success can only be fully understood in the context of complex feedbacks. The details provided in the current analyses offers an unprecedented level of insight into mechanisms that govern momentum and energy exchange in the the complex heterogeneous canopy environment, which are relevant for fire behavior assessments. Moreover, the analyses also shed light on the potential indicators of high intensity crowning behavior. To conclude, this work highlights the importance of accounting for the effects of vegetation management, fine-scale vegetation heterogeneity, winds, and turbulence on fire behavior when conducting prescribed burn operations and the success of initial attacks on wildland fires.

## Methods

### Fuel data

The fuel data were collected at the Eglin Air Force Base in Florida. Based on tree inventory data, three major species of trees were present, namely longleaf pines (*Pinus palustris*), common persimmon (*Diospyros virginiana L.*) and turkey oaks (*Quercus cerris*). Further details about the fuel data collection methods are described in^77^. The average tree canopy density (fine fuel) was 0.3 $\text {kg m}^{-3}$ for longleaf pines and persimmons. For turkey oaks, this value was 0.4 $\text {kg m}^{-3}$. The density of grass was 1.573 $\text {kg m}^{-3}$, with an average grass height of 0.5 m. The average litter height was 0.1 m and the litter load was 5.0 $\text {kg m}^{-3}$. Moreover, the density of grass was reduced and the litter density was increased below the trees due to canopy shading, using an exponential attenuation factor 5.0. Under the moist condition, the nominal fuel moisture was 133% for longleaf pines, 170% for persimmons, 200% for turkey oaks and 8% for grass and litter. The nominal fuel size was 0.0005 m for longleaf pines and persimmons and 0.0002 m for turkey oaks. Note that the persimmons and the turkey oaks usually comprised the midstory. In the dry season, the moisture for the turkey oaks were 15$\%$ and the persimmons were killed (omitted from the fuel complex in the model). Tree data were collected in three stages of management—‘no midstory’ where there are only 408 pines per hectares (1 hectare is equal to 10,000 square meters) , ‘well managed or sparse midstory’ where there are 408 pines, 551 persimmons and 44 turkey oaks per hectare; ‘unmanaged or dense midstory’ where there are 408 pines, 551 persimmons and 983 turkey oaks per hectare. The average height for the longleaf pines was 18 m and the average height for the midstory vegetation was about 11 m. It is also important to note that the fuel loading values (2.0–2.5 metric tons per hectare for midstory + understory) and 2.7–3.0 metric tons per hectare for overstory) are consistent with those observed both in the US Southeast (Longleaf and Loblolly pines)^78^ and the Southwest (Ponderosa pine and mixed conifer stands)^79^. So the observations in this work are deemed to reflect a wide range of conditions. Another important point to note is that the moist or dry conditions in fuel moisture are entirely driven by seasonality and not by other management efforts.

Since the purpose of these simulations was to isolate the effects of midstory fuel management on wildland fire behavior, surface fuel conditions were held unchanged. Varying the surface fuel moisture would impose additional variations in fire behavior unrelated to treatment evaluations—and it would further complicate the interpretation of the results. In addition, whether this level of midstory fuel treatment would be sufficient to make the surface fuel drier is not clear and the literature poses contrasting evidences. Whitehead et al.^15^ noted that forest thinning might lead to enhanced solar radiation, wind speed and near-surface temperature but did not find any significant changes in relative humidity or surface fuel moisture. Kalias and Kent^17^ lists several studies which reported fuel treatment effects on fuel moisture and fire behavior. Banerjee^18^ also offers a detailed review on this topic and summarizes these contrasting evidences. Some field experimental studies such as Bigelow and North^80^, Faiella and Bailey^81^ and Estes et al.^82^ have reported no appreciable changes in the surface fuel moisture post thinning. Bigelow and North^80^ argued that micrometeorological changes in the sub canopy environment post thinning can counter each other. An increase in wind speed after thinning could increase the turbulence driven mixing of the air above and below the canopy sub layer, thereby not allowing air temperature to increase or surface fuel moisture to decrease. On the other hand, some other studies such as Pook and Gill^83^, Weatherspoon et al.^84^ and Countryman^85^ noted that thinning could lead to a drier surface fuel layer, which could enhance fire intensity. Given these uncertainties, it is unclear at this stage if changes in the midstory fuel (without removing the entire crown) would lead to any appreciable changes in the surface fuel moisture. While more research is needed to address this topic, the surface fuel properties were held fixed in this study while the midstory fuel was varied across the six simulation cases in this study based on available data.

### Model description

Simulations are conducted using the HIGRAD/FIRETEC code developed at Los Alamos National Laboratory^86–88^. FIRETEC is a large-eddy simulation (LES) tool that can resolve atmospheric turbulence over three dimensional heterogeneous fuel distributions at spatial resolutions on the order of 2 m and can capture the spread, intensity and extent of burn area under different ignition conditions. FIRETEC simulates the movement of a wildfire by accounting for a few processes, such as the convective heating of fuel elements in front of a flame, the entrainment of cold air from the surroundings atmosphere, radiative heating and cooling of fuels and the drag experienced by the wind over vegetation canopy. The combustion of solids, which leads to chemical products and heat, is handled using a single compartment model without regards to chemical composition of the fuels. More specifically, it includes an evolution equation for the density of dry fuel as well as the density of water separately. A budget equation for the internal energy of the fuel that includes radiation, advection and energy exchange due to chemical reactions and evaporation of water tracks the temperature of the solid fuel elements. The evolution of the density and momentum of the gas phase are governed by the mass and momentum budgets of a fully compressible Navier Stokes equation. There is another budget equation for the internal energy of the gas phase which results in the potential temperature of the gas phase. It includes turbulent advection, turbulent diffusion as well as radiation effects and the energy exchange with the burning solids. Last but not the least, an advection diffusion equation tracks the evolution of oxygen. It is important to note that fine scale (below 2 m) processes are treated as sub grid scale processes. The sub grid scale variations of temperature, velocity and fine scale fuel features are parameterized.

### Simulations

Each simulation is conducted over a 400 m by 320 m (12.8 ha) domain with a grid resolution of 2 m by 2 m. The vertical extent of the domain is 550 m. The vertical grid resolution is non uniform and uses a grid stretching function in order to accommodate more grid points close to the surface within the vegetation canopy. There are 49 grid points in the vertical direction, 12 of which are within the canopy layer. The average grid spacing within the canopy layer is 1.8 m. The time step of each simulation is 0.02 s. Each simulation is spun up with a wind run for 3100 s (about 52 min) so that sufficient turbulence is generated and the wind field develops a steady state. Periodic boundary conditions are used on both sides of the domain. 80 processors are used in parallel for each set of simulations. An inlet wind profile with a value of 8 $\mathrm {ms}^{-1}$ at 30 m above ground level (AGL) is prescribed, which adjusts to the vegetation for the corresponding simulation during the wind run and follows a logarithmic profile above the zero plane displacement height. A free slip boundary condition is used at the top of the domain. The fire simulations start after the wind runs, with an ignition line, 4 m wide, 80 m long and offset by 80 m from the left edge of the domain along the wind direction (*x* axis) and centered perpendicular to the wind direction (*y* axis). Note that line ignitions are standard while investigating fundamental aspects of wildland fire behavior both in terms of experiments and simulations^46,87^. The target temperature of ignition is 1000 K and a ramp rate of 350 $\text {K s}^{-1}$ is used. Each fire simulation is run for 600 s (10 min).
